# Effects of the fat-tailed ewes' body condition scores at lambing on their metabolic profile and offspring growth

**DOI:** 10.5194/aab-63-183-2020

**Published:** 2020-06-24

**Authors:** Yathreb Yagoubi, Naziha Atti

**Affiliations:** Laboratoire de Productions Animales et Fourragères, INRA-Tunisia, University of Carthage, Rue Hédi Karray, 2049 Ariana, Tunisia

## Abstract

This experiment aimed to evaluate the effect of body condition score (BCS) of fat-tailed Barbarine ewes at lambing on their metabolic profile around parturition and lamb's growth. The experiment was carried out on 69 Barbarine ewes, divided into three groups according to BCS, which were inferior to 2, between 2 and 2.5 and superior to 2.5 for the thin, middle and fat group, respectively. Along the trial, all groups received the same dietary treatment based on hay, pasture and concentrate.

Birth weight (Bi-W), weights at 30 and 70 d (W30 and W70) and average daily gains (ADGs) of lambs were recorded. Metabolites were determined at late pregnancy and at the beginning of lactation. Ewes' BCS at lambing had no effect on lambs' Bi-W (P>0.05), which was 3.8, 3.8 and 3.9 kg, respectively, for thin, middle and fat groups. However, W30, W70 and ADG increased with a mother's BCS. A positive correlation between lamb growth parameters and ewe body weight and BCS at weaning was recorded. Energetic metabolites (glucose and triglycerides) and proteic metabolites (creatinine, total protein and urea) were similar among groups according to BCS but significantly different between pregnancy and lactation stages except triglycerides and urea. In conclusion, BCS may be used as dietary management tool during ewe lactation. With the transition from pregnancy to lactation, the content of some metabolites has changed irrespective of BCS; this aspect needs more investigations.

## 1 Introduction

The concept of body condition reflects the amount of body reserves, particularly fat, in the living animal (Kenyon et al., 2014). The body condition score (BCS) better indicates these reserves than live weight alone (Russel et al., 1969; Sanson et al., 1993; Atti and Bocquier, 2007). It has the potential to be a useful management tool for producers to increase animal performance, leading to decisions on when and how to practice nutrition supply to the whole flock or only a part, allowing assessment of animal nutrition level. Farmers may accept and use BCS as a management tool when they understand the benefits that it will provide to their production system. Therefore, it might be expected that ewes of lower BCS will display reduced reproductive performance in comparison with those of greater BCS (Atti et al., 2001; Kenyon et al., 2014). There is an optimum BCS for the flock at each stage of the production cycle. It was shown that females of different mammalian species such as sheep and goats mobilize their reserves in some critical physiological stages (pregnancy and lactation) in order to cover foetus needs and maintain their milk production (Chilliard et al., 1998). They also resort to mobilizing their reserves in the case of feed shortage, especially in dry areas, to meet their energy requirement and survive (Chilliard et al., 1998; Atti et al., 2004; Caldeira et al., 2007). This phenomenon reflects the capacity of ewes to adapt to different conditions while maintaining their vital functions. The fat-tailed sheep breeds like Barbarine are rustic and well adapted to the harsh conditions by using their body reserves (Atti et al., 2004). The amount of the tail fat presents a visible part of the body reserves; for the Barbarine breed, its weight varied from 1 to 4 kg (Atti et al., 2004). For this, a body condition score proper to fat-tailed sheep breeds has been developed (Atti and Bocquier, 2007).

There are many studies showing the relationship between BCS at mating and reproductive performance for thin-tailed (Griffiths et al., 2016) and fat-tailed sheep breeds (Atti et al., 2001). For the impact of ewe BCS at lambing on lamb growth, the research is abundant and with confounded conclusions for thin-tailed breeds (Caldeira et al., 2007; Kenyon et al., 2014; Corner-Thomas et al., 2015). However, for fat-tailed breeds having an additional body reserves site, results are scarce. From the bibliographic synthesis of Kenyon et al. (2014), ewe BCS could have no effects on lamb growth from birth to weaning and on weaning weight or have positive effects on these parameters. Given these variations between studies undertaken on thin-tailed ewes on a large spectrum of BCS values, the purpose of the current investigation was to study the effect of the fat-tailed Barbarine ewes' BCS at lambing on their lambs' growth, and we undertake this experiment in the limited spectrum of BCS. The effect of BCS on metabolic statute around parturition was also determined.

## 2 Material and methods

### 2.1 Ewes, diet and experimental design

The study was carried out at the experimental farm (Bourebiaa) of the National Institute of Agronomic Researches of Tunisia (INRAT) on 69 heads of fat-tailed Barbarine ewes. They were 3–4 years old, averaging 36.7+4.98 kg of body weight (BW) and judged healthy when submitted to mating. They were managed under semi-intensive conditions and naturally mated with fertile Barbarine rams. All animals received the same diet based on pasture, hay and concentrate during pregnancy and lactation (Table 1). The concentrate contained barley, soybean meal and vitamin–mineral supply (calcium carbonate, sodium chloride and phosphate) with 14 % of crude protein. Fresh water was at all times offered ad libitum. The breeding season of sheep extended from the beginning of July until the end of August, so the lambing continued from late November to January.

**Table 1:** Ewe diet during pregnancy and lactation.

Period	Ewe diet	Physiological stage
1 Jul–30 Sept	Cereal stubble	Pregnancy
1 Oct–15 Jan	Barley hay + concentrate (400 g ewe${}^{-1}$ d${}^{-1}$)	Late pregnancy, lambing and early lactation
15 Jan–15 Apr	Green pasture + barley hay	Lactation
15 Apr–30 Jun	Hay stubble + concentrate (200 g ewe${}^{-1}$ d${}^{-1}$)	Lactation + weaning

The BCS was regularly recorded for ewes every 15 d; it was taken at lumbar (LS) level and at caudal (CS) level according to Russel et al. (1969) and Atti and Bocquier (2007), respectively. Both LS and CS were determined by careful palpation; they were performed by two trained technicians and the adopted score value was determined in common agreement. Both BCS values were assessed on a five-point scale, with divisions of 0.25 points at each score. For each ewe, the calculated mean of both scores (LS and CS) was considered to characterize groups, and then ewes were divided into three groups according to the mean BCS at lambing:

- Thin group (25 ewes): BCS <2;
- Middle group (22 ewes): BCS between 2 and 2.5;
- Fat group (22 ewes): BCS >2.5.

### 2.2 Ewe body weight (BW) and lamb growth control

Ewes were weighed every 15 d. The BW and BCS were recorded from mating to lamb weaning. The lamb's number and birth weight (Bi-W) were recorded at birth. Then, lambs were weighed every 3 weeks until weaning at 4 months old. Weights at 30 and 70 d ages (W30 and W70) and average daily gain (ADG) were calculated by extrapolation as

- ADG${}_{\text{Bi–30}}$ = ADG between birth and 30 d
- ADG${}_{30-70}$ = ADG between 30 and 70 d

### 2.3 Blood sampling and haematological analyses

From all animals blood samples were collected through jugular venipuncture using heparinized Vacutainer tubes with no additive at two stages, 1 week before lambing (late pregnancy) and at the beginning of lactation (1 week after lambing). In order to keep the serum samples as fresh as possible, blood samples were centrifuged immediately after collection at 3000 g for 20 min; plasma samples were transferred into plastic tubes of 2 mL and frozen at -20 ${}^{\circ }$C for subsequent metabolite analysis. All haematological analyses were carried out using commercially available kits. For non-esterified fatty acids (NEFAs), the kits were not available. The dosage of glucose and triglyceride levels was carried out using phase kit supplied by Bio-Systems S.A. The dosage of total protein, urea and creatinine was conducted using the kit supplied by Phase Biomaghreb (Tunisia). The absorbance reading was performed by an ultra-visible spectrophotometer (Milton Roy, France). Glucose and triglycerides absorbance was read at 500 nm; however, creatinine, total protein and urea absorbance were read at 492, 546 and 590 nm, respectively.

**Figure 1:**
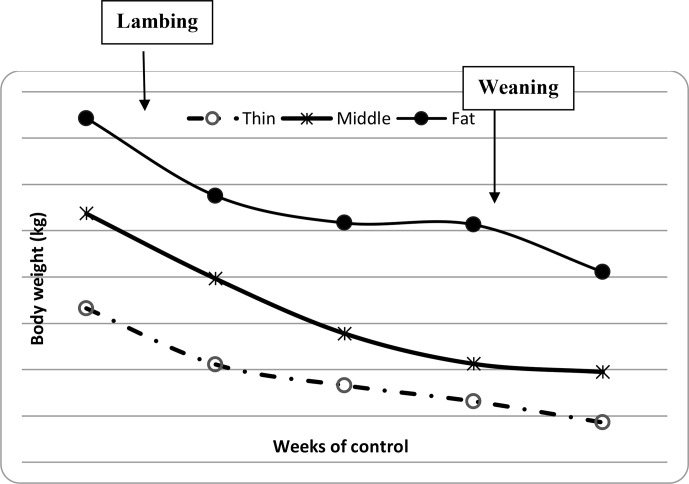
Variation of ewe body weights from lambing to weaning period.

**Figure 2:**
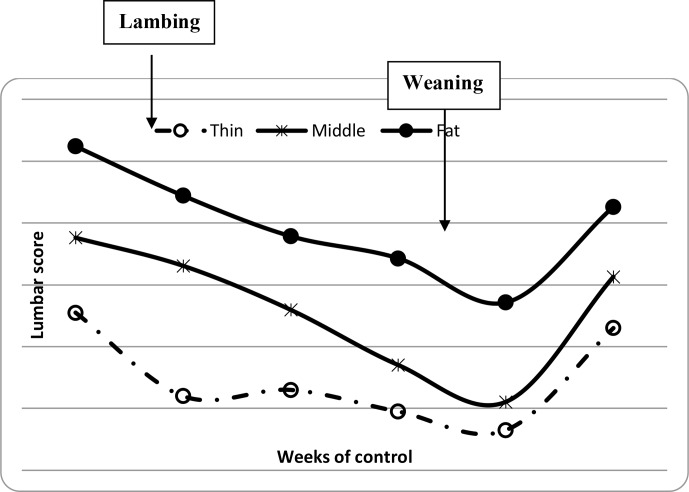
Variation of ewe lumbar scores from lambing to weaning period.

**Figure 3:**
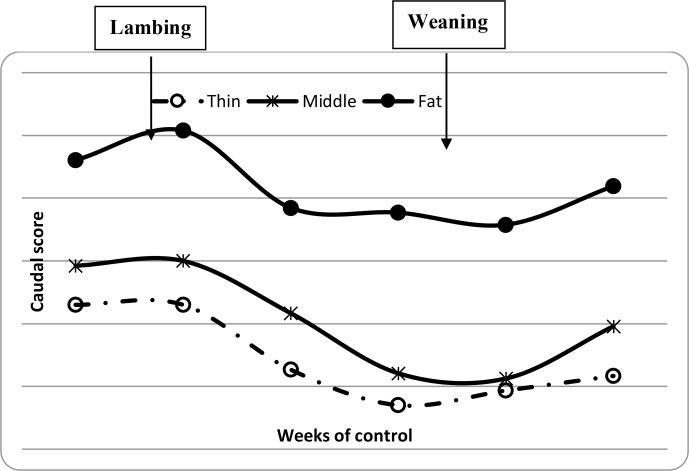
Variation of ewe caudal scores from lambing to weaning period.

### 2.4 Statistical analysis

A one-way ANOVA was used to test the effect of a ewe's body condition score (thin, middle or fat) on a lamb's growth parameters using GLM (general linear model procedure of S.A.S. Institute, 1989). The differences between groups was compared by Duncan's test. In addition, the correlation between the different parameters was determined using the correlation procedure of a statistical analysis system (SAS). Data of ewe metabolites during the two physiological stages of measurements (pregnancy and lactation) were analysed using the MIXED procedure for repeated measures of SAS. The analyses were performed with a ewe's body condition score as a between-subject fixed effect, a physiological stage as a within-subject effect and a random animal effect as subject (experimental unit). For all the tests, the level of significance was 0.05.

## 3 Results and discussion

### 3.1 Ewe body weight, body condition score and body reserves mobilization

The descriptive statistics for ewe BCS (LS and CS) and BW (kg) are reported in Table 2. The BW of ewes at lambing was significantly different (P=0.001) among groups. The mean BW of ewes was 32.8, 36.7 and 41 kg for thin, middle and fat groups, respectively. Ewes with the higher BCS (fat) were heavier than ewes from thin and middle groups by 8.2 and 4.3 kg, respectively. This result confirmed that BW increased with improving BCS; this positive relationship between BW and BCS was previously shown (Atti et al., 1995; Kharrat and Bocquier, 2010; Sejian et al., 2015). Then, ewes' BW and BCS decreased between lambing and weaning (Figs. 1, 2 and 3) until reaching low body scores, being a reflection of body reserve mobilization to cover the lamb's needs along the suckling period (Chilliard et al., 1998). During this period between lambing and weaning, the body weight lost was 2.8 kg in both middle and fat groups, being significantly (p=0.01) higher for them than thin ones (Table 3), which decreased their body weight slightly (1.1 kg), seeing that they are meagre and they have no reserves to mobilize. This significant decrease in BW, LS and CS during the suckling stage is the consequence of the physiological state, which is common for all females of different mammalian species and breeds who lose body reserves in the beginning of lactation (Chilliard et al., 1998; Beker et al., 2010). Furthermore, for the current study the ewes were undernourished; they were forced to mobilize body reserves to ensure higher milk production for their lambs (Atti et al., 2004; Beker et al., 2009). These results confirmed other studies on goats and sheep. It was shown that fat goats lost weight (1.84 kg) and BCS during lactation, while the thin ones increased (+0.43 kg) their BW (Kharrat and Bocquier, 2010). Also, fat ewes at lambing lost more weight (4.2 vs. 3.5 kg) and BCS (0.64 vs. 0.34) than thin ones (Atti et al., 1995). After lamb weaning, all ewes begin to increase their BW, LS and CS (Figs. 1, 2 and 3) by using their diets to replenish their reserves lost during pregnancy and lactation.

**Table 2:** Descriptive statistics for ewes' body weight (BW), lumbar and caudal scores at lambing.

	Mean	SD	Minimum	Maximum
Body weight (kg)	36.68	4.98	25.8	50.8
Lumbar condition score	1.20	0.34	0.5	2.25
Caudal condition score	3.57	0.86	1.5	5.0

**Table 3:** Body weight (BW), lumbar (LS) and caudal (CS) scores of ewes between lambing and weaning.

	Lambing				Weaning			
	Thin	Middle	Fat	p value	Thin	Middle	Fat	p value
BW	32.8${}^{c}$	36.7${}^{b}$	41.0${}^{a}$	0.001	31.7${}^{c}$	33.9${}^{b}$	38.2${}^{a}$	0.001
LS	0.84${}^{c}$	1.25${}^{b}$	1.6${}^{a}$	0.001	1.1${}^{b}$	1.2${}^{b}$	1.5${}^{a}$	0.001
CS	2.9${}^{c}$	3.4${}^{b}$	4.5${}^{a}$	0.001	2.6${}^{b}$	2.9${}^{b}$	4.1${}^{a}$	0.001
Mean BCS	1.9${}^{c}$	2.3${}^{b}$	3.0${}^{a}$	0.001	1.8${}^{c}$	2.1${}^{b}$	2.8${}^{a}$	0.001
	BW and BCS variation between lambing and weaning							
	Thin		Middle		Fat		p value	
Δ BW	-1.1${}^{b}$		-2.8${}^{a}$		-2.8${}^{a}$		0.015	
Δ LS	0.26${}^{b}$		-0.05${}^{a}$		-0.1${}^{a}$		0.001	
Δ CS	-0.3		-0.5		-0.4		0.9	
ΔBCS	-0.1		-0.2		-0.2		0.87	

Mean BCS: mean between LS and CS; ΔBW: the variation in BW between lambing and weaning; ΔLS: the variation in LS between lambing and weaning; ΔCS: the variation in CS between lambing and weaning. Different superscripts means differences between groups.

### 3.2 Lamb growth

The lamb growth parameters are shown in Table 4. Irrespective of a ewe's BCS, the lamb's Bi-W was similar for all groups averaging 3.8 kg (P>0.05). Similar results, where ewes' BW at lambing did not affect lamb growth parameters, were previously reported for the same breed (Atti et al., 2004) and other breeds (Kenyon et al., 2012; Karakus and Atmaca, 2016). However, others works reported that ewes from other breeds with higher BW or BCS at lambing had lambs with higher birth weights (Clarke et al., 1997).Then, the result of the current study can be explained by the fact that thin ewes, even with low BCS, have drawn on their body reserves during pregnancy to support the requirement of the conception. The ability of the Barbarine ewes to achieve pregnancy with acceptable performance even in underfeeding conditions has been shown (Atti et al., 2004). For rustic breeds, the birth weight of the lamb may be considered as a breed-defining characteristic, which is less determined by the body condition score of the ewe mother.

**Table 4:** Lamb growth parameters.

Group	Thin	Middle	Fat	p value
Bi-W	3.8	3.8	3.9	0.3
W30	7.81${}^{b}$	8.40${}^{\mathrm{ab}}$	9.25${}^{a}$	0.009
W70	12.0${}^{b}$	12.63${}^{b}$	14.45${}^{a}$	0.003
ADG1	148${}^{b}$	165${}^{\mathrm{ab}}$	204${}^{a}$	0.02
ADG2	105${}^{b}$	106${}^{b}$	131${}^{a}$	0.007

Bi-W: birth weight; W30: lamb weight at 30 d; W70: lamb weight at 70 d; ADG1: average daily gain between birth weight and W30; ADG2: average daily gain between W30 and W70. Different superscripts means differences between groups

The relationship between a ewe's BCS at different physiological stages and a lamb's birth weight was widely examined. Some studies outlined a significant effect (Corner-Thomas et al., 2015; Sejian et al., 2015), while other reported no effect (Aliyari et al., 2012). It is probable that this difference is due to differences in the timing of BCS measurement, ewe nutrition and particularly breed characteristics and ability to mobilize body reserves.

For W30, W70 and both ADGs (ADG${}_{\text{Bi–30}}$, ADG${}_{30-70}$), lambs from the fat group had significantly higher values than those of the thin one, while lambs of the middle group had intermediate values. These differences may be explained by the higher milk production, which is the result of a higher reserve mobilization of fat ewes compared to thin and middle ones (Atti et al., 1995). This phenomenon was reported for thin-tailed (Caldeira et al., 2007) and fat-tailed breeds (Atti et al., 2004) where the ewes in higher body condition used their body reserves to cover their energy requirement, even in undernutrition, to maintain a high level of milk production to suckle their lambs. In the same context, Kharrat and Bocquier (2010) showed that thin goats increased their energy intake to maintain milk yield near to that of fat ones. Karakuş and Atmaca (2016) recorded similar results, although not statistically significant; they showed that lambs issued from ewes with the highest BCS (3.5) had higher live weights, between 30 and 120 d of age, than lambs from BCS 2.5 and BCS 3.0 ewes. For lambs of all groups in the current study, the ADG${}_{30-70}$ was significantly lower than the ADG${}_{\text{Bi–30}}$. This phenomenon may be the result of the low nutrient availability in the second stage, which did not provide energy leading to the same growth as that of the mother milk. The ADG${}_{\text{Bi–30}}$ demonstrated the maternal capacity to rear its offspring, while the ADG${}_{30-70}$ reflects the own potential growth of lambs since the ewes at this stage (30–70 d) had no more reserves as in the first use (Bi-30 d) to produce milk for their offspring. This tendency was reported for the same breed for which the lamb's ADG${}_{30-70}$ was frequently lower than the ADG${}_{10-30}$ in correct and undernutrition conditions (Atti et al., 2004).

Positive and significant correlations were recorded between W30, W70, ADG${}_{\text{Bi–30}}$ and ADG${}_{30-70}$ on the one hand and BW, LS and CS at lambing on the other (Table 5).The lamb W30, W70 and both ADGs were significantly correlated with ewes' BW, LS and CS at weaning (Table 6). The correlation coefficients varied between 0.414 and 0.645. Significant correlations were recorded between the BW variation (ΔBW) between lambing and weaning and lamb W30 and W70. This result should be taken into account in the operation of culling ewes where they lost more BW and BCS during suckling to permit more growth for their offspring and should be maintained in the flock even if they are have poor BW or BCS at lamb weaning. In the other situation, it was reported that BCS had no significant effect on a lamb's weaning weight (Kenyon et al., 2011). The different conclusions among studies could result from differences in nutrition during pregnancy and lactation (Karakus and Atmaca, 2016) or from the feeding level and feed quality offered (Kenyon et al., 2014). Also the breed characteristics could affect behaviour; in fact, genetic and maternal factors influenced foetal development and account for over 30 % of the variation in birth weight (Johnston et al., 2002).

### 3.3 Ewe metabolic profile

The results of metabolic profile according to a ewe's BCS around parturition were shown in Table 7. Concentrations of blood metabolites for ewes in this study were consistent with the normal range for healthy sheep. In the current study, all metabolites were not affected by the ewes' BCS. Similar results were found, where ewes with a different BCS did not affect glucose level (Jalilian and Moeini, 2013). However, Caldeira et al. (2007) recorded different metabolic status for ewes with a different BCS with lower glycaemia for thin (BCS between 1 and 2) than fat animals (BCS between 3 and 4). In addition, Mazur et al. (2009) showed lower values for plasma glucose and triglycerides for undernourished ewes. While Pesántez-Pacheco et al. (2019) reported that sheep with higher BCS during pregnancy and postpartum showed a greater triglycerides concentration than did sheep with lower BCS. The creatinine level was similar for all ewes, which is in contrast with results reported by Caldeira et al. (2007) where it increased in case of undernutrition and with low body condition scores (1 and 2). Similarly, total protein and urea were unaffected by a ewe's BCS. However, in previous studies, BCS appears to influence protein metabolism after lambing or calving where sheep and dairy cows with higher BCS showed an increase in urea level than thinner ones (Karapehlivan et al., 2007).

**Table 5:** Correlation coefficients among ewes' body weight (BW), lumbar (LS) and caudal (CS) at lambing and lamb growth parameters.

	Bi-W	W30	W70	ADG1	ADG2
BW at lambing	0.120	0.621	0.645	0.514	0.490
p value	0.324	0.001	0.001	0.001	0.001]
LS at lambing	0.143	0.471	0.507	0.514	0.414
p value	0.244	0.001	0.001	0.001	0.001]
CS at lambing	0.093	0.450	.544	0.534	0.559
p value	[0.562	0.004	0.001	0.001	0.001]
W30	0.219	1.0	0.904	0.801	0.526
p value	[0.137	–	0.001	0.001	0.001]
W70	0.300	0.904	1.0	0.756	0.839
p value	0.04	[0.001	–	0.001	0.001]

Bi-W: birth weight; W30: lamb weight at 30 d; W70: lamb weight at 70 d; ADG1: average daily gain between birth weight and W30; ADG2: average daily gain between W30 and W70.

**Table 6:** Correlation coefficients among ewes' body weight (BW), lumbar (LS) and caudal (CS) at weaning and lamb growth parameters.

	Bi-W	W30	W70	ADG1	ADG2
BW at weaning	0.254	0.584	0.612	0.530	0.471
p value	0.068	[0.001	0.001	0.001	0.001]
LS at weaning	0.206	0.398	0.410	0.392	0.318
p value	0.142	[0.001	0.001	0.001	0.012]
CS at weaning	0.176	0.487	0.547	0.572	0.52
p value	0.211	[0.001	0.001	0.001	0.001]
Δ BW	-0.800	0.324	0.311	0.212	0.219
p value	0.001	[0.009	0.014	0.095	0.086]
Δ LS	-0.802	0.217	0.216	0.276	0.194
p value	0.001	[0.08	0.09	0.028	0.13]
Δ CS	0.006	-0.072	-0.007	-0.077	0.053
p value	0.962	[0.65	0.96	0.63	0.74]

W30: lamb weight at 30 d; W70: lamb weight at 70 d; ADG1: average daily gain between birth weight and W30; ADG2: average daily gain between W30 and W70.

**Table 7:** Ewe metabolites (mmol L${}^{-1})$ according to lambing body condition score and the physiological stage.

	Ewe group (G)			Physiological stage (PS)		p value		
	Thin	Middle	Fat	Pregnancy	Lactation	G	PS	G × PS
Glucose	0.48	0.57	0.62	0.73${}^{a}$	0.37${}^{b}$	0.32	0.001	0.68
Triglycerides	0.47	0.41	0.53	0.51	0.42	0.44	0.18	0.89
Total protein	62.2	60.98	61.3	58.7${}^{b}$	64.5${}^{a}$	0.59	0.001	0.68
Creatinine	6.81	6.68	8.02	5.27${}^{b}$	9.08${}^{a}$	0.33	0.001	0.58
Urea	0.27	0.29	0.29	0.29	0.27	0.48	0.27	0.68

Different superscripts means differences between groups.

In the current study, the similarity of metabolite concentrations irrespective of the BCS may be explained by the same ewe management conditions and/or the high rusticity, resilience and adaptation of Barbarine ewes and generally the fat-tailed breeds to harsh conditions. Especially for creatinine, which is an indicator of protein or muscle catabolism, similar concentrations means that even with low BCS ewes are able to cover the foetus requirement and do not need to use their muscles.

Irrespective of the ewe's BCS, for the energetic metabolites, the physiological stage (late pregnancy and lactation) significantly affected (p=0.001) the content of glucose; however, triglycerides level was unaffected. The results found for glucose level are in agreement with those reported in other works (Caldeira et al., 2007) for pregnant ewes (0.42–0.76 mmol L${}^{-1}$) and for lactating ones (0.41–0.65 mmol L${}^{-1}$; Dubreuil et al., 2005). The decrease in glucose concentration for all groups in lactation compared to pregnancy could be explained by the higher demand, related to needs, of glucose in postpartum than that during pregnancy (Block et al., 2001). These results corroborate other studies for goats suggesting that glucose is critical molecule for meeting a goat's nutritional requirement during lactation (Cepeda-Palacios et al., 2018). In fact, this phenomenon is related to the increase in milk production, which involves mobilization of glucose for the synthesis of milk lactose (McNeill et al., 1998), which was also confirmed for cattle (Bach, 2012). The triglycerides concentration between pregnancy and lactation (0.51 and 0.42 mmol L${}^{-1}$, respectively) was comparable to usual values reported by Mollereau et al. (1995). There was a slight decrease in triglycerides level, but it was not significant. This phenomenon may be explained by the transition of triglycerides in the milk of lactating ewes because the milk fat is composed essentially of triglycerides (Nazifi et al., 2002). Then, triglycerides in the blood are fuel sources that are consumed when energy requirements increase during pregnancy and lactation (Nazifi et al., 2002; Caton and Hess, 2010; Pesántez-Pacheco et al., 2019). These responses of the energetic metabolites through suckling are the results of lipid mobilization (Mazur et al., 2009) to cover the high-energy needs during this physiological stage (Chilliard et al., 1998).

Concerning the proteic metabolites, creatinine and total protein were significantly affected by the ewe's physiological stage, while urea level was unaffected. The lactating ewes had a higher creatinine concentration than pregnant ones (9.08 vs. 5.27 mmol L${}^{-1}$, respectively). Yokus et al. (2006) recorded the same tendency but without significant difference. Moreover, Roubies et al. (2006) reported a significant influence of the reproductive stage on creatinine concentration and attributed this difference to the development of the foetus musculature. The total protein concentration during lactation was significantly higher than that during pregnancy. Jelinek et al. (1985) recorded the same tendency with same value during lactation progress (from 58.7 to 64.5 mmol L${}^{-1})$. However, Celi et al. (2008) found that total protein level was significantly lower after parturition than in pregnant goats. It was shown that the decrease in the blood protein for goats is due to its removal from the blood stream in order to support mammary secretion after parturition (Chen et al., 1998).The urea concentrations are within the norms of Ndoutamia and Ganda (2005) for pregnancy (0.20-0.30 mmol L${}^{-1}$) and for lactation (0.32+0.17 mmol L${}^{-1}$) but were not affected by the physiological stage. This parameter is related to the importance of protein intake in the diet and especially the protein efficiency for small ruminants (Friot and Calvet, 1973). Indeed, for the current study the diet level and its protein content were similar in late pregnancy and the beginning of lactation. The proteic metabolites of ewes were in the normal range for healthy sheep; then, these results indicate that the nutritional management of the ewes was appropriate regardless of the normal changes related to the physiological stages.

**Table 8:** Correlation coefficients among lamb growth parameters and metabolic profile parameters.

	Bi-W	W30	W70	ADG1	ADG2
Glucose 1${}^{a}$	0.101	0.215	0.102	0.266	-0.079
p value	[0.412	0.092	0.431	0.036	0.541]
Triglycerides 1	-0.051	-0.197	-0.161	-0.237	-0.044
p value	[0.677	0.124	0.214	0.063	0.733]
Creatinine 1	-0.028	0.136	0.113	0.140	0.065
p value	[0.817	0.289	0.381	0.276	0.614]
Total protein 1	-0.276	0.124	0.132	0.145	0.160
p value	[0.054	0.414	0.392	0.341	0.298]
Urea 1	0.108	0.059	0.092	0.137	0.074
p value	[0.457	0.696	0.550	0.367	0.631]
Glucose 2${}^{b}$	0.069	0.070	0.174	0.044	0.248
p value	[0.582	0.595	0.189	0.737	0.060]
Triglycerides 2	0.128	-0.141	0.101	-0.035	0.349
p value	[0.313	0.284	0.446	0.791	0.007]
Creatinine 2	0.004	0.238	0.228	0.224	0.153
p value	[0.970	0.071	0.087	0.090	0.252]
Total protein 2	-0.025	-0.010	0.003	0.046	0.080
p value	[0.865	0.944	0.985	0.769	0.618]
Urea 2	0.050	0.049	0.037	-0.107	-0.001
p value	[0.732	0.748	0.811	0.488	0.995]

${}^{a}$: Pregnancy. ${}^{b}$: lactation

The correlation test between metabolic profile and lamb growth parameters showed a positive and significant correlation between ADG${}_{\text{Bi–30}}$ and glucose (r=0.266; P=0.036). However, a negative and significant correlation between ADG${}_{\text{Bi–30}}$ and triglycerides was shown (r=-0.337; P=0.063, Table 8). Zywicki et al. (2016) showed that foetal plasma glucose and triglycerides levels were directly related to foetal weight (P<0.0001), while Hu et al. (1990) found that total weight of lamb born was negatively related to plasma glucose concentration (r=-0.22; P<0.01). The ADG${}_{30-70}$ tended to be related to the glucose and triglycerides levels during lactation, and the correlations were positive. Total protein level during pregnancy was inversely correlated with lamb birth weight, and the correlation was significant (r=-0.276; P=0.054). However, in previous studies (Addah and Karikari, 2008), the relationship between total protein and birth weight was nearly linear (r=0.93; P<0.05). It was reported that maternal body protein can only serve as a major source of protein for supporting visceral organ metabolism and foetal growth without significant effect on maternal body under moderate levels of undernutrition but not under chronic nutritional conditions (Robinson et al., 1999). Creatinine concentration during lactation tended to be positively correlated with W30, W70 and ADG${}_{\text{Bi–30}}$, but no relationship between lamb growth parameters and urea level was observed as previously shown (Hu et al., 1990). Lamb growth parameters were not related to creatinine level during pregnancy, while Zywicki et al. (2016) reported that foetal plasma creatinine levels were inversely related to foetal weight (P<0.0001).

## 4 Conclusions

This study showed that fat-tailed ewes mobilize their reserves during pregnancy to cover the conception requirements to reach lambing even with low BCS and produced lambs with similar birth weight. However, the lamb growth rate and their weight at 30 and 70 d were higher for offspring of ewes with middle and high BCS. Since the BCS can be useful as a dietary management tool after lambing, at this stage, a high diet level is required to meet suckling needs in general but especially for those who are thinner than middle and high BCS sheep. These results did not shown any relationship between a ewe's BCS at lambing and their metabolic profile; this aspect would be studied with more frequent blood sampling.

## Data Availability

The original data of the paper are available upon request to the corresponding author.
